# Design and Optimization of a Stationary Electrode in a Vertically-Driven MEMS Inertial Switch for Extending Contact Duration

**DOI:** 10.3390/s17030527

**Published:** 2017-03-07

**Authors:** Qiu Xu, Zhuo-Qing Yang, Bo Fu, Yan-Ping Bao, Hao Wu, Yun-Na Sun, Meng-Yuan Zhao, Jian Li, Gui-Fu Ding, Xiao-Lin Zhao

**Affiliations:** 1 National Key Laboratory of Science and Technology on Micro/Nano Fabrication, School of Electronic Information and Electrical Engineering, Shanghai Jiao Tong University, Shanghai 200240, China; xuqiu@sjtu.edu.cn (Q.X.); Cecilia_Sun@sjtu.edu.cn (Y.-N.S.); zhaomy@sjtu.edu.cn (M.-Y.Z.); lijian19930405@126.com (J.L.); xlzhao@sjtu.edu.cn (X.-L.Z.); 2 Huaihai Industrial Group Co., Ltd., Changzhi 046012, China; fuhbo_406@163.com (B.F.); baoyanping304@163.com (Y.-P.B.); df11g25t@126.com (H.W.)

**Keywords:** inertial micro-switch, surface-micromachining, stationary electrode, geometrical parameters, contact time

## Abstract

A novel micro-electro-mechanical systems (MEMS) inertial microswitch with a flexible contact-enhanced structure to extend the contact duration has been proposed in the present work. In order to investigate the stiffness *k* of the stationary electrodes, the stationary electrodes with different shapes, thickness *h*, width *b*, and length *l* were designed, analyzed, and simulated using ANSYS software. Both the analytical and the simulated results indicate that the stiffness *k* increases with thickness *h* and width *b*, while decreasing with an increase of length *l*, and it is related to the shape. The inertial micro-switches with different kinds of stationary electrodes were simulated using ANSYS software and fabricated using surface micromachining technology. The dynamic simulation indicates that the contact time will decrease with the increase of thickness *h* and width *b*, but increase with the length *l*, and it is related to the shape. As a result, the contact time decreases with the stiffness *k* of the stationary electrode. Furthermore, the simulated results reveal that the stiffness *k* changes more rapidly with *h* and *l* compared to *b*. However, overlarge dimension of the whole microswitch is contradicted with small footprint area expectation in the structure design. Therefore, it is unreasonable to extend the contact duration by increasing the length *l* excessively. Thus, the best and most convenient way to prolong the contact time is to reduce the thickness *h* of the stationary electrode while keeping the plane geometric structure of the inertial micro-switch unchanged. Finally, the fabricated micro-switches with different shapes of stationary electrodes have been evaluated by a standard dropping hammer system. The test maximum contact time under 288 g acceleration can reach 125 µs. It is shown that the test results are in accordance with the simulated results. The conclusions obtained in this work can provide guidance for the future design and fabrication of inertial microswitches.

## 1. Introduction

Recently, MEMS inertial microswitches based on surface micromachining technology have been attracting much attention due to many advantages, such as lower cost, less power consumptions, larger volume production, smaller size, better performance, and more functionality than conventional mechanical inertial switches fabricated with precision machining processes [1]. Therefore, they are widely used in an array of applications, such as accessories, automotive electronics and remote monitoring (RMON) [2,3,4]. Compared to the accelerometer, the inertial microswitch is both a sensor and an actuator. Firstly, this purely mechanical trigger in the inertial switch is superior to the accelerometer because they can avoid the risk of mis-operation from electromagnetic interference [5]. Secondly, it is not necessary to measure the continuously varying acceleration signals in some long-lifetime or small scale systems because of the limited power supply, where accelerometers are not suitable because of the necessity for a continuous power supply [6]. However, the inertial microswitch can overcome the above shortcoming and be applied to some long-lifetime systems, since the inertial microswitch, as a sort of passive device, draws no power until an acceleration event happens [7,8,9,10].

In traditional designs, both the movable electrode and the stationary electrode in the inertial microswitch are usually rigid, which will result in an extremely short contact duration. However, this extremely transient contact time will become a significant obstacle for subsequent signal processing if the microswitch is applied in an integrated circuit [10,11,12,13]. In order to facilitate the signal processing and enhance the contact effect, Wang et al. designed a laterally-driven inertial microswitch using two cantilever beams as the fixed electrode, which can reduce the contact-bouncing effect and prolong the contact time [14]. Then, Qihuan Zhang et al. reported a laterally-driven inertial microswitch with the double-stair shape flexible cantilever beam as the movable electrode for prolonging the contact time [15]. Deng et al. designed the inertial microswitch with double flexible electrodes based on the silicon bulk micromachining process, which can further improve the contact effect [16]. Several kinds of vertically-driven inertial microswitches with flexible contacts have been proposed and fabricated in our previous works [5,6,17,18,19]. In these cases, one elastic crossbeam was regarded as the stationary electrode instead of rigid electrode, which indicates that the contact duration has been extended to some extent. However, the test contact time was less than 60 µs. A convenient way to further prolong the contact time is to decrease the stationary electrode’s stiffness. Unfortunately, the influence of geometric parameters of the stationary electrode on the contact time in the inertial microswitch was hardly studied in detail. In the present work, two different designs of elastic cross beams with holes were introduced into the vertically-driven inertial microswitch as the stationary electrode for extending the contact duration. The effect of shape, thickness (*h*), width (*b*), length (*l*) of the stationary electrode on the contact duration will be fully analyzed by analytical and finite-element simulation methods. Finally, the fabricated inertial micro-switch devices will be also evaluated by a standard drop hammer system.

## 2. Analysis, Design, and Simulation

### 2.1. Theoretical Analysis of the Stiffness of the Stationary Electrode

**Figure 1:**
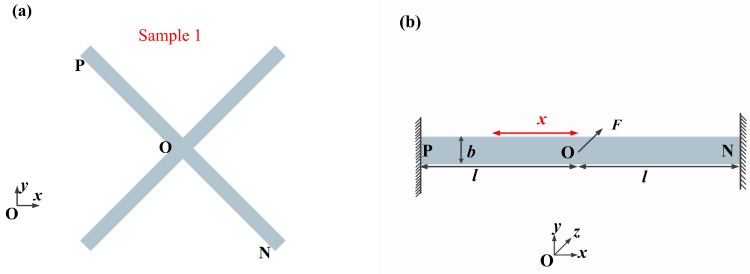
(**a**) Two-dimensional sketch of the stationary electrode in sample 1; and (**b**) the plane structure diagram of the cantilever beam in sample 1.

Sample 1 is the originally-designed stationary electrode as shown in Figure 1a, which includes two linear structure cantilever beams. In consideration of the geometric symmetry of the structure, it can be seen from Figure 1b that the stationary electrode can be simplified as one linear structure cantilever beam (PN) to analyze, the stiffness *k* of the stationary electrode in sample 1 is equal to the total sum stiffness of two linear cantilever beams (2 $k_{beam}$). The thickness, width, and length of linear cantilever beam are *h*, *b*, and *l*, respectively. Both ends (P and N) of the cantilever beam is constrained to be zero, and the center point (O) is applied by a concentrated force *F* in *z* direction as shown in Figure 1b. *x* is in *x* direction along the length of cantilever beam. The total length of the cantilever beam (PN) is approximately 2*l*. According to the data [20], the maximum deflection of the center point O under the concentrated force *F* can be obtained by Equation (1):

(1) $$\delta =\frac{Fl^{3}}{24EI}$$

The stiffness $k_{beam}$ of the linear cantilever beam (AB) in *z* direction can be expressed by Equation (2) according to Hooke’s Law:

(2) $$k_{beam}=\frac{F}{\delta }=\frac{24EI}{l^{3}}$$

where *E* and *I* are the Young’s modulus and the bending moment of inertia of the linear cantilever beam, respectively. *I* can be defined as:

(3) $$I=\frac{bh^{3}}{12}$$

Substituting Equation (3) into Equation (2), the stiffness *k* of the stationary electrode in sample 1 can be written as:

(4) $$k=2k_{beam}=\frac{4Ebh^{3}}{l^{3}}$$

**Figure 2:**
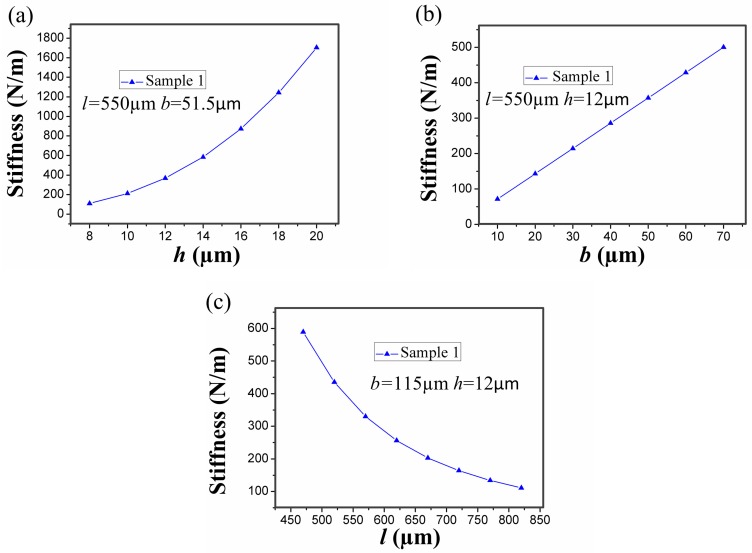
Dependence of analytical stiffness *k* of the stationary electrode on its geometric parameters (*h*, *b*, *l*): (**a**) the stiffness *k* versus the thickness *h*; (**b**) the stiffness *k* versus the width *b*; and (**c**) the stiffness *k* versus the length *l*.

The analytical stiffness *k* of the stationary electrode in sample 1 is proportional to *b* and $h^{3}$, while being inversely proportional to $l^{3}$. Figure 2a–c shows that the stiffness *k* of the stationary electrode in sample 1 varies with *h*, *b*, and *l*. It reveals that *h* and *l* more easily affects the stiffness *k* compared to *b*. However, the dimension of the fabricated microswitch cannot be too large because of the expected small footprint in the structure design. Therefore, the length *l* cannot be too long. Thus, the best way to control the stiffness *k* is to change the thickness *h* instead of *b* and *l*. The analytical models neglect the deformation from beam elongation, beam shortening, and shear. This assumptions is reasonable because these deformations are smaller than the deformation from bending moment and the deflection from torsion. The limitations of the analytical models neglect a series of holes in the cantilever beam.

**Figure 3:**
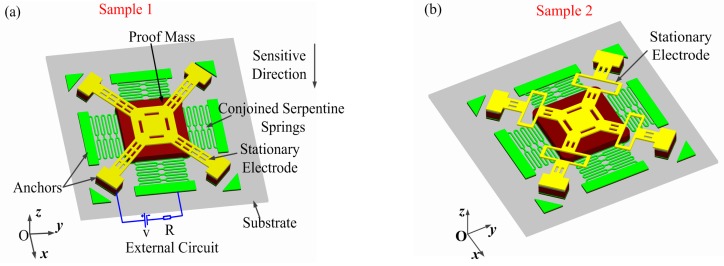

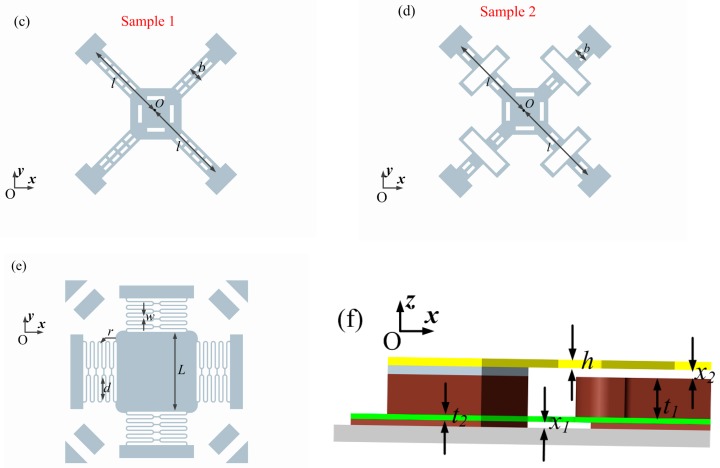
Three-dimensional view of the micro-switch (**a**) in sample 1 and (**b**) in sample 2; 2D sketch of the stationary electrode (**c**) in sample 1 and (**d**) in sample 2; (**e**) top view of the whole structure; and (**f**) side view of one-half structure.

The mouth-shape crossbeam in sample 2 (see Figure 3d) is used as the stationary electrode. Considering that the calculation of deflection in sample 2 must consider more factors than the one in sample 1 because of its complicated struture, the calculated process of the analytical solution in sample 2 is very fussy and difficult. Therefore, only the analytical solution in sample 1 is provided in this paper.

### 2.2. Structure Design

Two vertically-driven inertial microswitches with differently-shaped crossbeams as the stationary electrode for extending contact duration are designed. It can be seen from Figure 3a that the inertial microswitch of sample 1 is mainly made up of two parts. The first part is one elastic cross beam as the stationary electrode. A series of holes in the stationary electrode can facilitate the final device’s release. Additionally, it can reduce the stiffness of the elastic crossbeam and effectively extend the contact duration. The second part is the suspended proof mass which connects with four serpentine springs. The previous report [10,11] and simulations in the present work show that the contact time of sample 1 is less than 100 µs. In order to further prolong the switch-on time, one redesigned inertial micro-switch of sample 2 with a mouth-shaped cross beam as the stationary electrode is shown in Figure 3b. The geometric parameters of the stationary electrode in samples 1 and 2 are shown in Figure 3c,d. The thickness (*h*), width (*b*), and length (*l*) of the stationary electrode are chosen as variables. Figure 3e,f show the top view and side views of the inertial microwitch. The main geometric parameters are marked in Table 1.

### 2.3. Simulation

When the mass-spring system is shocked by an acceleration in its sensing direction (*z* direction), the sensing movable system can be described as follows [21]:

(5) $$m\ddot{x}+c\dot{x}+kx=ma(t)$$

where *m* stands for the proof mass, x is the displacement, *c* is the damping coefficient, *k* is the whole system, stiffness and *a*(*t*) represents the applied acceleration.

**Figure 4:**
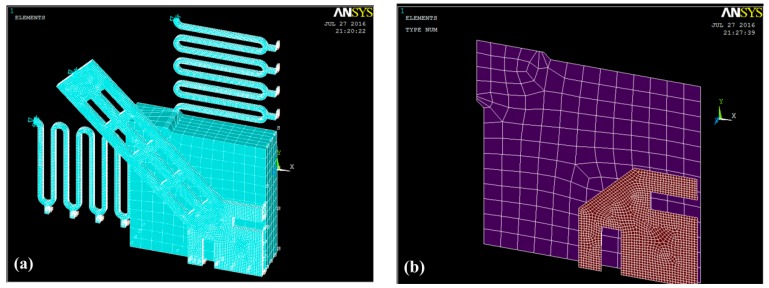
(**a**) ANSYS finite element simulation model of the inertial microswitch in sample 1; (**b**) its contact pair in sample 1.

One-quarter of the inertial microswitch will be simulated by the commercial ANSYS software in order to save computing time, as shown in Figure 4a. The symmetric boundary conditions were applied onto the symmetric surface of the model. The end sections of the fixed electrode and the two suspended serpentine springs away from the proof mass are constrained to zero in all degrees of freedom. The serpentine springs and the stationary electrode have the finest mesh in order to model the dynamic contact process more accurately. While, for the proof mass, only the region near the springs has the finer mesh grid for saving the computing time. Transient analysis has been introduced to gain the displacement curves changing with the time. In this simulation, we selected SOLID 186, TARGE170, and CONTA174 as the element types with the SWEEP method of meshing the model. The contact pair between the stationary electrode and the proof mass is shown in Figure 4b. Electroplated metal nickel (Ni) is chosen as the main structure material. In light of our previous experimental results, the properties of electroplated nickel are as follows: Young’s modulus is 171.5 GPa, Poisson’s ratio is 0.3, and density is 8.96 g·cm^−3^ [22].

#### 2.3.1. Dependence of the Contact Time on Different Shape of the Stationary Electrode

**Figure 5:**
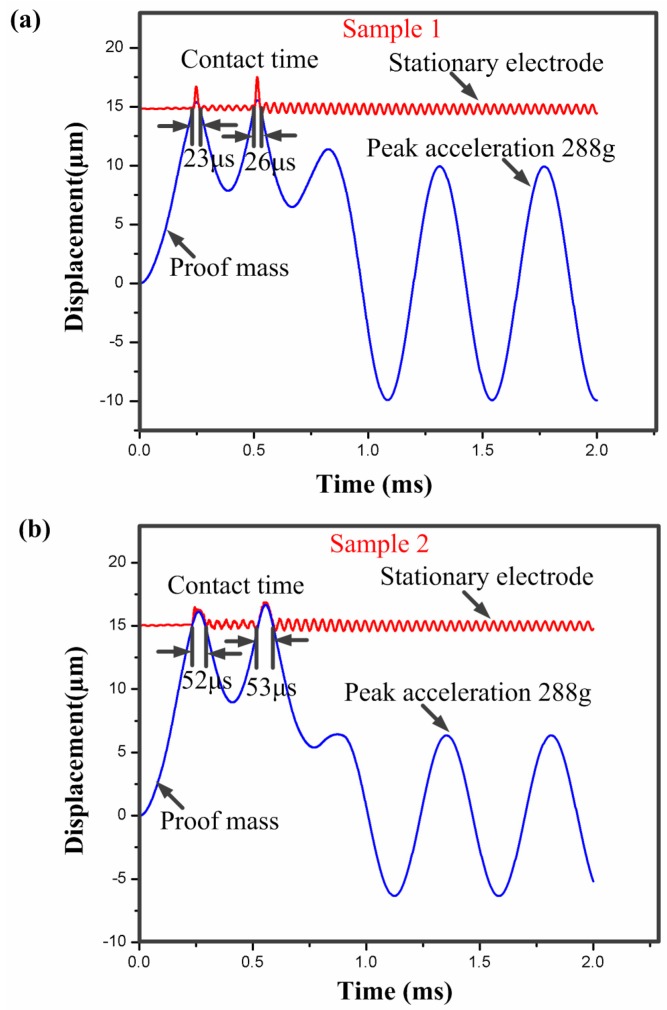
Dynamic response curves of the inertial micro-switch under the applied acceleration amplitude of 288 g. (**a**) Sample 1; and (**b**) Sample 2.

**Figure 6:**
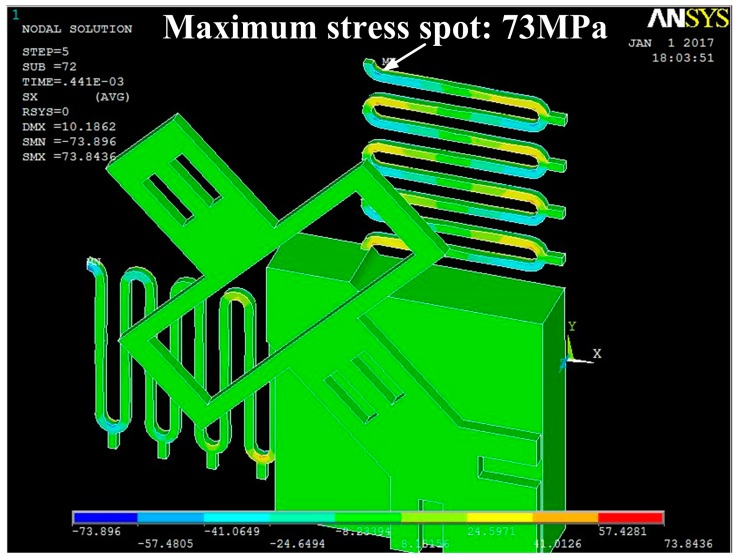
The maximum stress distribution of the serpentine springs and the stationary electrode of sample 1 under the applied acceleration 288 g.

For a microswitch device, the shape of the stationary electrode is a crucial factor which can affect the contact time. In the designed inertial microswitch, samples 1 and 2 have the same geometric parameters (*h*, *b*, *l*) of the stationary electrode. In order to study the effect of the stationary electrode’s shape on the contact time, the same acceleration $a_{0}$ (288 g) applied to the two FE models in samples 1 and 2. Figure 5 shows the simulated dynamic response displacement curves of the proof mass and the contact time of samples 1 and 2 under the acceleration 288 g is 26 µs and 53 µs, respectively. The simulated result in Figure 5 shows that the contact time relates to the shape of the stationary electrode. This reason is mainly that the shape of the stationary electrode determine the stiffness of the stationary electrode compliant cantilever beam. The contact time decreases with the increase of the stiffness of elastic cross beam. It can be seen from Figure 2 that the stiffness of the mouth-shaped stationary electrode in sample 2 is smaller than the one in sample 1. Thus, the contact time is longer in sample 2. This reveals that the shape of the stationary electrode is also a key factor to prolong the contact time. Figure 6 shows the stress distribution of the serpentine springs and the stationary electrode in sample 1 reaching the maximum displacement when the acceleration 288 g was applied to the microswitch. It is evident that the maximum stress spot (73 MPa) is smaller than the yield strength (317 MPa) of electroplated nickel. Therefore, it is a purely elastic deformation during the whole dynamic contact process and the springs cannot break.

#### 2.3.2. Effect of Thickness of the Stationary Electrode on the Contact Time

**Figure 7:**
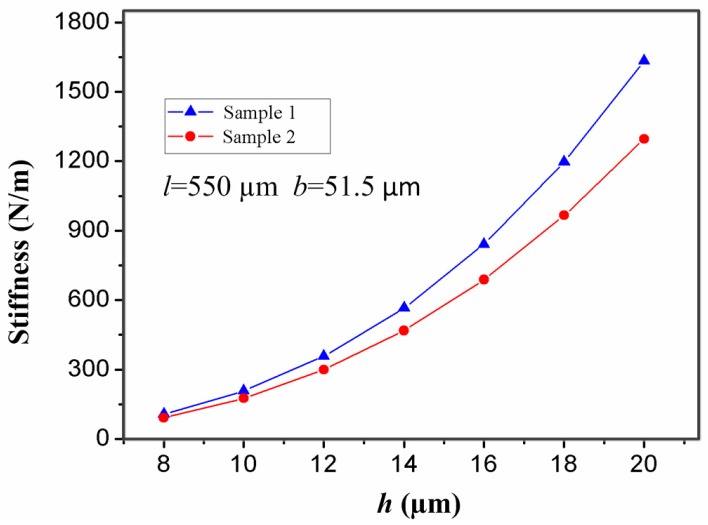
The stiffness of the stationary electrode in samples 1 and 2 versus its thickness (*h*).

**Figure 8:**
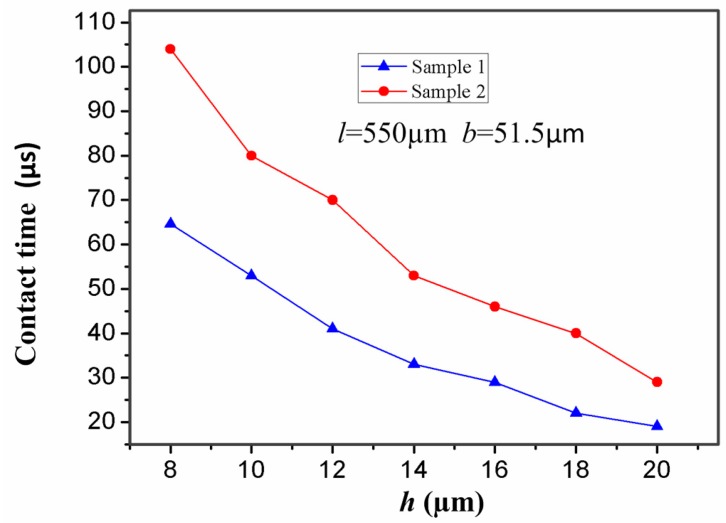
Dependence of contact time in samples 1 and 2 on the thickness (*h*) of the stationary electrode under the applied acceleration 288 g.

In addition, the thickness of the stationary electrode has also a great impact on the contact time. For the inertial microswitch, decreasing the thickness of the stationary electrode is a key method to extend contact duration. In order to study the impact of the thickness (*h*) of the stationary electrode on the contact time, the width (*b*) and length (*l*) of the stationary electrode in samples 1 and 2 are invariant and set as 51.5 µm and 550 µm, respectively. The stiffness of the two designs of elastic cross cantilever beams was simulated by ANSYS software [23]. The simulated stiffness of the stationary electrode *k* = $\frac{F}{\delta }$ can be obtained according to Hooke’s law. Figure 7 shows that the stiffness of the stationary electrode increases with the thickness, and the stiffness in sample 1 is larger than the one in sample 2 for the same thickness. When samples 1 and 2 are shocked by the same acceleration 288 g, the contact time of the inertial microswitch as a function of the thickness (*h*) is shown in Figure 8. The simulated results indicate that the contact time decreases with the increase of the thickness (*h*). That is to say, the contact time of the inertial micro-switch device decreases with the increase of the stiffness of the stationary electrode. It can be seen from the plot in Figure 8 that the contact time in sample 2 is larger than the one in sample 1 since the stiffness of the stationary electrode in sample 2 is smaller compared to the one in sample 1. It is evident that the contact time can be controlled by changing the thickness of the stationary electrode.

#### 2.3.3. Effect of Width of the Stationary Electrode on the Contact Time

**Figure 9:**
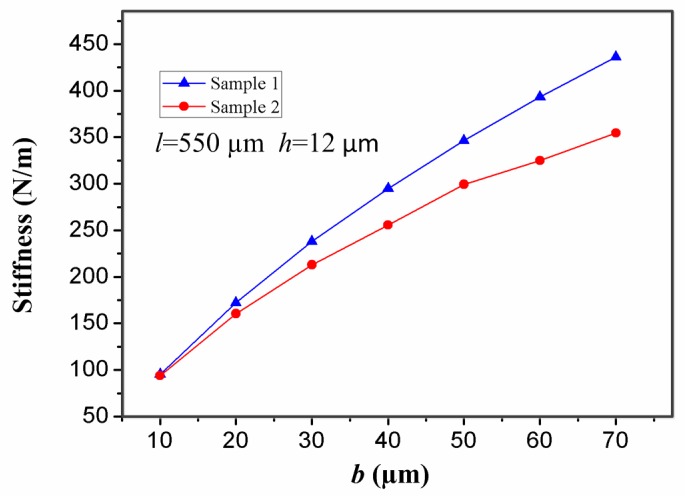
Effect of the width (*b*) of the stationary electrode in samples 1 and 2 on the stiffness of the stationary electrode when the thickness (*b*) and the length (*l*) of the stationary electrode are set as 12 µm and 550 µm, respectively.

**Figure 10:**
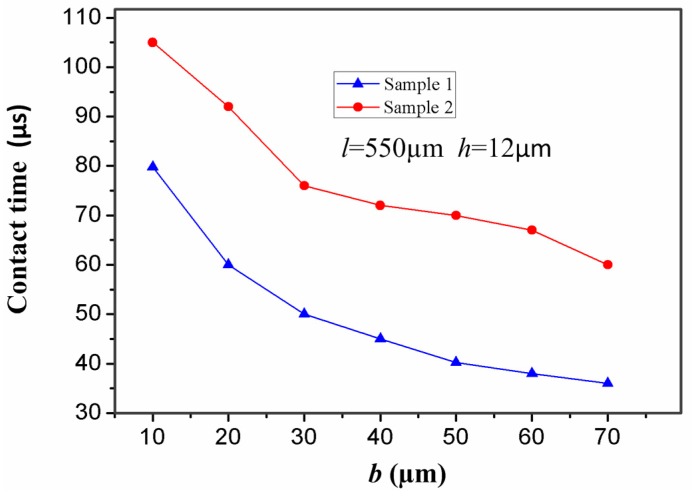
Dependence of contact time in samples 1 and 2 on the width (*b*) of the stationary electrode under the applied acceleration 288 g.

It is well known that the width (*b*) of the stationary electrode also has a great impact on the contact time. For the inertial micro-switch, minimizing the stiffness of the stationary electrode is a crucial way to prolong the contact time. One of the best ways to lessen the stiffness of the stationary electrode is to lessen its width (*b*). It can be seen from Figure 9 that the stiffness of the stationary electrode increases with the width (*b*). Figure 10 shows the contact time-width (*b*) curves of samples 1 and 2 under the applied acceleration 288 g. The contact time in samples 1 and 2 decreases with the increase of the width (*b*) of the stationary electrode. That is to say, the contact time decreases with the increase of the stiffness of the stationary electrode. It is evident that the contact time can be controlled by changing the width (*b*) of the stationary electrode. It can be seen from Figure 10 that the contact time in sample 2 is larger than the one in sample 1 since the stiffness of the stationary electrode in sample 2 is smaller.

#### 2.3.4. Effect of Length of the Stationary Electrode on the Contact Time

**Figure 11:**
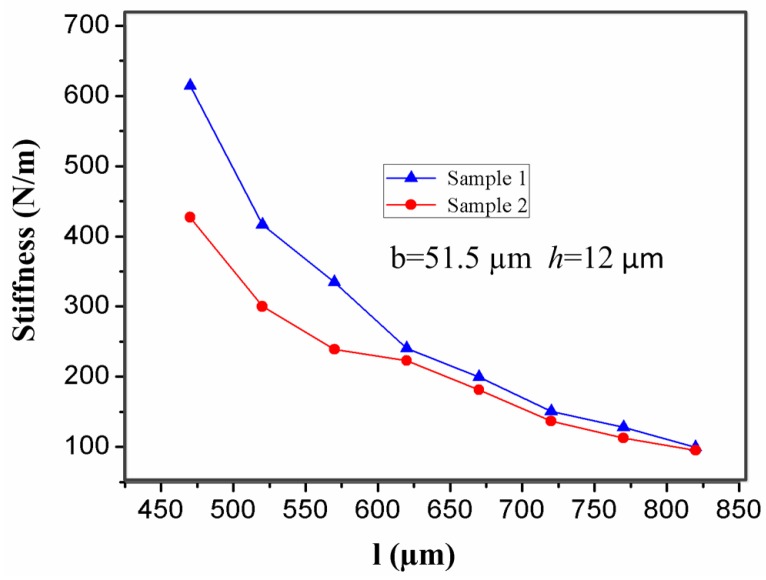
Effect of the length (*l*) of the stationary electrode on its stiffness when the width (*b*) and the thickness (*h*) of the stationary electrode are 51.5 µm and 12 µm, respectively.

**Figure 12:**
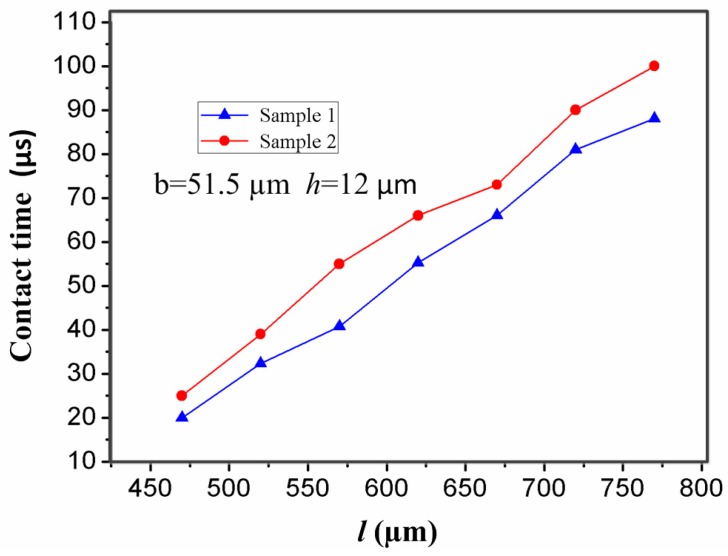
Dependence of contact time in samples 1 and 2 on the length (*l*) of the stationary electrode under the applied acceleration 288 g.

For a microswitch device, length (*l*) of the stationary electrode is a key factor affecting the contact time of the inertial micro-switch. In the designed inertial microswitch, samples 1 and 2 have the same geometric parameters (*h*, *b*) of the stationary electrode, but length (*l*) is variable. Figure 11 shows the stiffness-length curves of the stationary electrode in samples 1 and 2 under the acceleration 288 g. It can be seen that the stiffness of the stationary electrode decreases as the length increases. At the same time, Figure 12 shows that the contact time increases with the length of the stationary electrode. It can be seen from Figure 12 that the contact time in sample 2 is larger than the one in sample 1, since the softer the stationary electrode is, the longer the contact time is. However, overly large dimensions of length are not a satisfactory choice since the dimension of the whole microswitch cannot be too large, considering the miniature structure design.

The thickness, width, and length of the stationary electrode in samples 1 and 2 are key factors affecting the stiffness of the stationary electrode. Firstly, it reveals that the stiffness of the stationary electrode increases with the thickness and width of the stationary electrode. Secondly, the stiffness decreases with the increase of the length. Thirdly, the stiffness in sample 1 is larger than the one in sample 2 for the same geometric parameters (*h*, *b*, *l*) of the stationary electrode. Therefore, the stiffness relates to the shape of the electrode. In conclusion, the contact time decreases as the stiffness of the stationary electrode increases. It has been proven that the simulated stiffness in sample 1 is in agreement with the above analytical calculation in sample 1. The slight deviation may be attributed to the following reasons: Firstly, the center cross part of the simulated model shown in Figure 3c is an area while the center cross part of the analytical model in Figure 1a is simplified as a point(O); secondly, the simulated model has a series of holes to reduce the stiffness.

## 3. Micro-Fabrication

**Figure 13:**
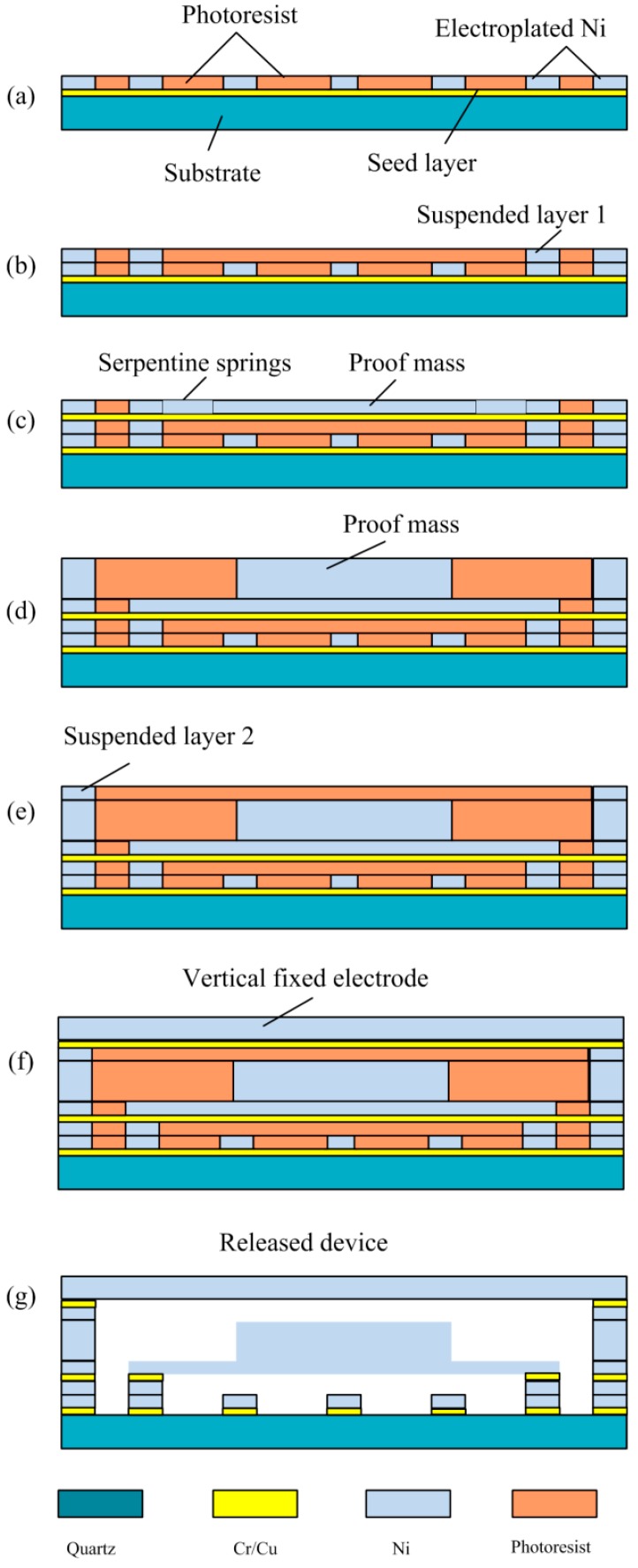
Main fabrication process sketch of the vertically-driven inertial microswitch.

The prototype of the vertically-driven inertial microswitch was fabricated by low-cost nickel-electroplating technology. In this work, we selected PR AZP4620 as the mold and sacrificial layer. We used electroplating nickel as the main structure material due to its excellent mechanical property. The electroplating-nickel residue can be reduced by optimizing the electroplating conditions. In the present work, the electroplating nickel’s condition can be listed as follows: temperature 45 °C, current density 125 mA/cm^2^, and pH 4.0. The entire micro-fabrication process as shown in Figure 13 can be listed as follows:

(a) The Cr/Cu was sputtered on the insulated quartz substrate as the first seed layer. We can electroplate the anchors of the spring and an array of raised strips after 10 µm photoresist (PR, AZP4620) spining-on and patterning.
(b) The anchors which suspended the spring and the stationary electrode were electroplated in Ni metal. Therefore, the first suspending layer gap (*x*_1_) can be formed.
(c) The four serpentine springs were electroplated after the second chromium/copper seed layer was sputtered.
(d) The proof mass was electroplated up to its required thickness (70 µm) in terms of multiple electroplated technology.
(e) A gap *x*_2_ (15 µm) between the proof mass and the stationary electrode was formed after the anchors which suspend the stationary electrode were electroplated higher (15 µm) than the proof mass.
(f) The stationary electrode was electroplated up to the required thickness.
(g) The photoresist was dissolved in 7% sodium hydroxide solution. Then the completed released device which was rinsed in isopropyl alcohol was dried in the oven to avoid stiction.

**Figure 14:**
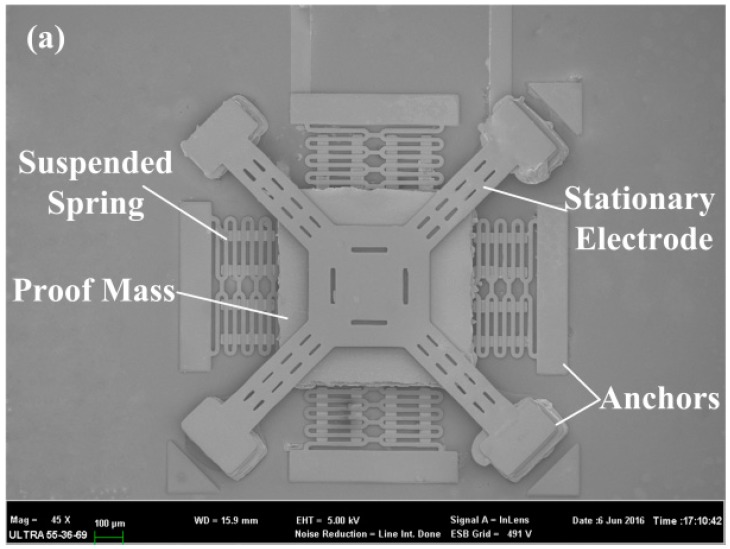

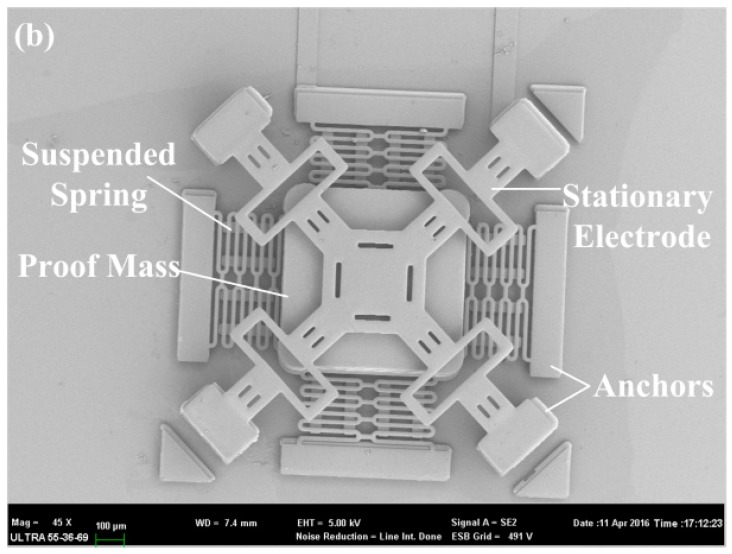
SEM photos of the fabricated inertial microswitch (**a**) with a cross beam as the stationary electrode in sample 1 and (**b**) with a mouth-shape cross beam as the stationary electrode in sample 2.

The inertia microswitch was sealed using an all-metal cap that was fabricated by ultraviolet Lithographie Galvanoformung Abformung technology. The dimension of the packaged single device by epoxy adhesive bonding is about 2.4 mm × 1.9 mm × 1.1 mm, which is smaller than existing devices [6]. The stationary electrode and the movable electrode are leaded from the sealed cap at the bottom of the insulation layer, respectively. The encapsulated device substrate is cut into a lot of discrete components. The scanning electron micrographs (SEM) of the completed released microswitches (2.4 mm × 1.9 mm × 1.1 mm) in samples 1 and 2 are shown in Figure 14a,b.

## 4. Experiment Test

**Figure 15:**
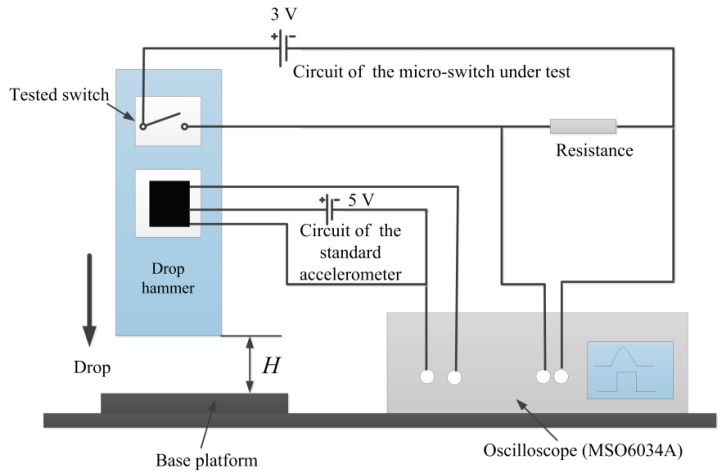
Schematic diagram of the tested setup for the fabricated microswitch.

It can be seen from Figure 15 that the drop hammer system was used to test the fabricated device. The standard accelerometer, ADXL-193, which has a sensitivity of 8 mV/g and was made by Analog Devices Inc. in Norwood, MA, USA, can be applied to the calibration. Simultaneously, the output signal was collected by an Agilent 6000 oscilloscope which was made in Agilent Technologies Co. Ltd., Santa Clara, CA, USA. Firstly we can fix the test device and the accelerometer on the dropping table, whose sensing direction is vertical to the base platform. The drop hammer could be dropped freely onto the base platform from different pre-set height *H* as shown in Figure 14, generating different accelerations which were applied to the test microswitch. It is evident that the amplitude of the acceleration changes with the pre-set height *H*. The drop hammer can drop freely from a different pre-set height *H*, and an array of heights correspond to a series of acceleration amplitudes. The amplitude of the acceleration increases with *H*. The acceleration signal and the trigger signal can be detected by the oscilloscope when the test microswitch switches on.

When the drop hammer falls from the required height of 52 cm, a half sine wave acceleration with the amplitude of 288 *g* which was applied to the test device could be generated. The oscilloscope records the test results of the fabricated micro-switches with different stationary electrode’s geometric parameters *h*, *b*, and *l*.

**Figure 16:**
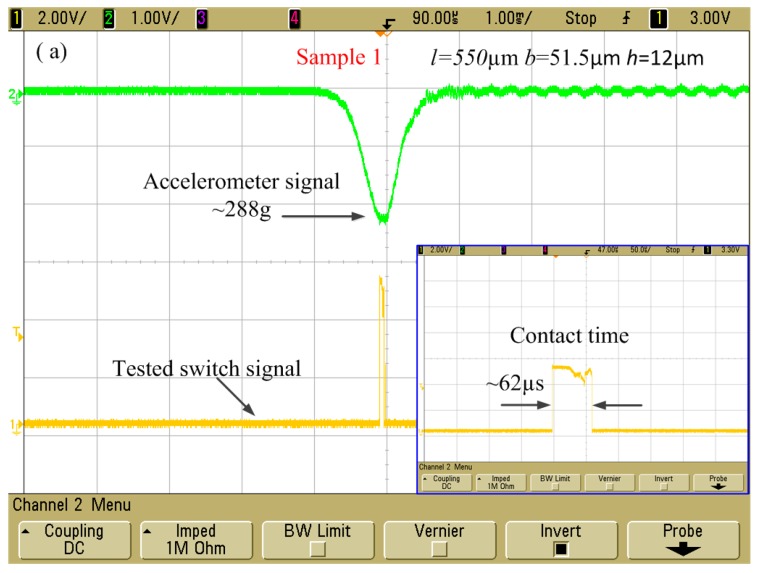

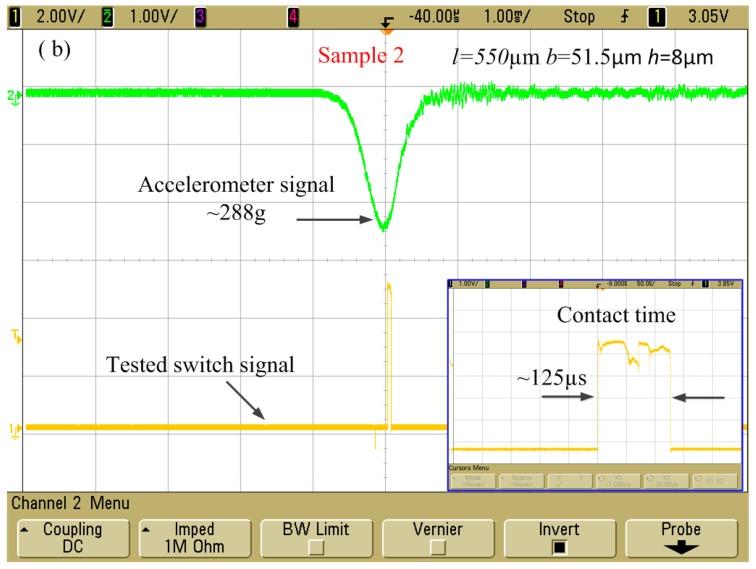
Test results of the inertial microswitches under the shock acceleration amplitude of 288 g: (**a**) The geometric parameters (*l*, *b*, *h*) of the stationary electrode are 550 µm, 51.5 µm, and 12 µm in sample 1; (**b**) The geometric parameters (*l*, *b*, *h*) of the stationary electrode are 550 µm, 51.5 µm, and 8 µm in sample 2.

**Figure 17:**
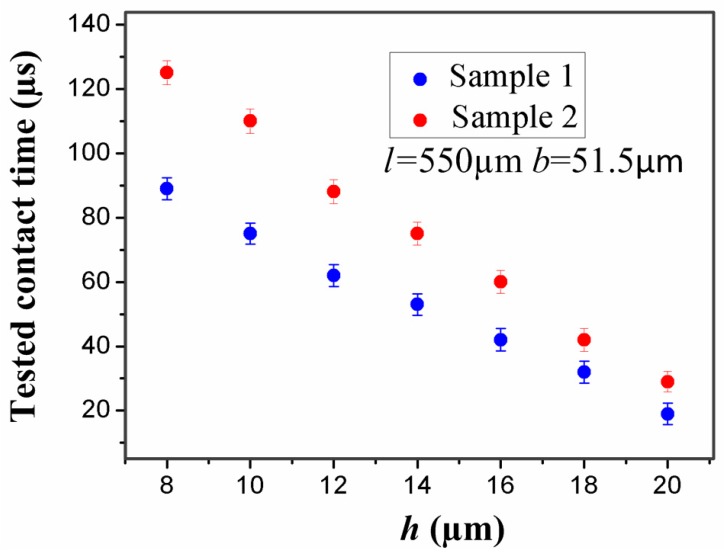
Dependence of the test contact time on the thickness (*h*) of the stationary electrode when the acceleration amplitude is 288 g.

The original design inertial micro-switch in sample 1 (*h* = 12 µm, *b* = 51.5 µm, and *l* = 550 µm) was shocked by the applied acceleration of 288 g. As is shown in Figure 16a, the test contact time was about 62 µs, which is much longer than the one reported elsewhere. In order to further extend the contact duration, the redesigned inertial microswitch in sample 2 (*h* = 8 µm, *b* = 51.5 µm, and *l* = 550 µm) can be triggered with 125 µs contact time under the applied acceleration of 288 *g* as shown in Figure 16b. The stationary electrode in the first case is one elastic crossbeam, and its stiffness is high. Therefore, the contact time is short. The redesigned stationary electrode in the second case is a mouth-shape crossbeam with a low stiffness, and it can further prolong the switch-on time. Figure 17 indicates a series of test contact time with a different thickness of the stationary electrode. Each thickness (*h*) test result is the average value of three samples’ repeated experiments. According to the former analytical and the simulation results, it reveals the thickness *h* easily affects the stiffness *k* and the plane geometric structure cannot be changed. The simulated results indicate that the contact time decreases with the increase of the thickness (*h*). That is to say, the contact time of the inertial microswitch device decreases with the increase of the stiffness of the stationary electrode. It can be seen from the plot in Figure 7 that the contact time in sample 2 is larger than the one in sample 1. The test results, as shown in Figure 16, have an agreement with these simulated results. The test contact time decreases with the increase of the thickness of the stationary electrode as shown in Figure 17. The simulated result as shown in Figure 8 is in accordance with these test results. The little difference between the simulated results and test results is attributed to the fabrication imperfection and the deviation between the applied acceleration and the ideal half-sine wave acceleration [10].

## 5. Conclusions

A MEMS inertial microswitch with an elastic crossbeam stationary electrode for extending the contact duration has been designed, simulated, and fabricated on a quartz substrate using surface micromachining technology. The simulation reveals that the stiffness of the stationary electrode relates to the shape of the stationary electrode and increases with the width (*b*) and thickness (*h*), but decreases with the increase of length (*l*). Meanwhile, the contact time decreases with the increase of the stiffness of the stationary electrode. Both the analytical results and the simulated results indicate that among all of the factors, *h* and *l* more easily affect the stiffness *k* compared to *b*. However, the dimension of the whole microswitch cannot be too large considering the miniature structure design. Therefore, it is not a workable way to prolong the contact time by increasing the length *l* too much. It is evident that to reduce the thickness *h* will not affect the plane geometric dimension of the inertial microswitch. Thus, the best and most convenient way to extend the contact duration is to reduce the thickness *h*. Finally, the fabricated microswitch has been tested by a drop hammer system. The redesigned inertial micro-switch of sample 2 (*h* = 8 µm, *b* = 51.5 µm, and *l* = 550 µm) can realize the contact time of 125 µs when a half-sine wave acceleration of 288 g is applied, which would facilitate the subsequent signal processing in its application system. The test results indicate that the contact time increases when decreasing the stiffness of the stationary electrode, which is in accordance with the simulated ones. These conclusions obtained in this work will provide some guidance for the future design and fabrication of the inertial microswitch.

## Figures and Tables

**Table 1:** Main geometric parameters of the designed device.

Components	Geometric Parameters	Value (µm)
Proof mass	Thickness *t*_1_ app:ds:length	80
	Length *L*	620
gaps	*x*_1_	5
	*x*_2_	15
spring	Thickness *t*_2_	10
	Width *w*	12
	Inside diameter *r*	10
	Length *d*	180
Fixed electrode	Thickness *h*	variable
	Length *l*	variable
	Width *b*	variable

## References

[1] Wei M., Zohar Y., Wong M. (2003). Design and characterization of inertial-activated electrical micro-switches fabricated and packaged using low-temperature photoresist molded metal-electroplating technology. J. Micromech. Microeng..

[2] Wycisk M., Tönnesen T., Binder J., Michaelis S., Timme H.-J. (2000). Low-cost post-CMOS integration of electroplated microstructures for inertial. Sens. Actuators A Phys..

[3] Michaelis S., Timme H.J. (2000). Additive electro-plating technology as a post-CMOS process for the production of MEMS acceleration-threshold switches for transportation applications. J. Micromech. Microeng..

[4] Zimmermann L., Ebersohl J.P., Le Hung F.L., Berry J.P., Baillieu F., Rey P., Diem B., Renard S., Caillat P. (1995). Airbag application: A microsystem including a silicon capacitive accelerometer, CMOS switched capacitor electronic and true self-test capability. Sens. Actuators A Phys..

[5] Cai H., Ding G., Yang Z., Su Z., Zhou J., Wang H. (2008). Design, simulation and fabrication of a novel contact-enhanced MEMS inertial switch with a movable contact point. J. Micromech. Microeng..

[6] Cai H., Yang Z., Ding G., Wang H. (2009). Development of a novel MEMS inertial switch with a compliant stationary electrode. IEEE Sens. J..

[7] Kuo J.C., Kuo P.H., Lai Y.T., Ma C.W., Lu S.S., Yang Y.J.J. (2013). A passive inertial switch using MWCNT-hydrogel composite with wireless interrogation capability. J. Micro-Electromech..

[8] Ma C.W., Huang P.C., Kuo J.C., Kuo W.C., Yang Y.J. (2013). A novel inertial switch with an adjustable acceleration threshold using an MEMS digital-to-analog converter. Microelectron. Eng..

[9] Michaelis S., Timme H.J., Wycisk M. (2000). Acceleration threshold switches from an additive electroplating MEMS process. Sens. Actuators A Phys..

[10] Zhang H., Zhu J., Zhu Z., Jin Y., Li Q., Jin G. (2013). Surface-plasmon-enhanced GaN-LED based on a multilayered M-shaped nano-grating. Opt. Express.

[11] Zhu J., Zhang H., Zhu Z., Lib Q., Jina G. (2014). Surface-plasmon-enhanced GaN-LED based on the multilayered rectangular nano-grating. Opt. Commun..

[12] Currano L.J., Yu M., Balachanran B. (2010). Latching in a MEMS shock sensor: Molding and experiments. Sens. Actuators A Phys..

[13] Xu Q., Yang Z., Fu B., Li J., Wu H., Zhang Q., Sun Y., Ding G., Zhao X. (2016). A surface-micromachining-based inertial micro-switch with compliant cantilever beam as movable electrode for enduring high shock and prolonging contact time. Appl. Surf. Sci..

[14] Zhang Q., Yang Z., Xu Q., Wang Y., Ding G., Zhao X. (2016). Design and fabrication of a laterally-driven inertial microswitch with multi-direction constraint structures for lowering off-axis sensitivity. J. Micromech. Microeng..

[15] Deng K.F., Su W.G., Li S., Zhang Y.T., Chen G.Z., Zhang W., Wan S., Li L., Jiang S.B., Zheng H. A novel inertial switch based on nonlinear-spring shock stop. Proceedings of the IEEE Transducer 2013 Conference.

[16] Wang Y., Feng Q., Wang Y., Chen W., Wang Z., Ding G., Zhao X. (2013). The design, simulation and fabrication of a novel horizontal sensitive inertial microswitch with low g value based on MEMS micromachining technology. J. Micromech. Microeng..

[17] Yang Z., Ding G., Chen W., Fu S., Sun X., Zhao X. (2007). Design, simulation and characterization of an inertia microswitch fabricated by non-silicon surface micromachining. J. Micromech. Microeng..

[18] Yang Z., Ding G., Cai H., Zhao X. (2008). A MEMS inertial switch with bridge-type elastic fixed electrode for long duration contact. IEEE Trans. Electron..

[19] Cai H., Yang Z., Ding G., Zhao X. (2008). Fabrication of a MEMS inertial switch on quartz substrate and evaluation of its threshold acceleration. Microelectron. J..

[20] Beam Formulas with Shear and Moment Diagrams. http://www.awc.org/pdf/codes-standards/publications/design-aids/AWC-DA6-BeamFormulas-0710.pdf.

[21] Meirovitch L. (1986). Elements of Vibration Analysis.

[22] Tang J., Wang H., Liu R., Li X., Zhang Z., Yao J., Ding G. (2010). A directly strain measuring method for electroplated nickel micro-tensile test. J. Microsyst. Technol..

[23] ANSYS Inc.. http://www.ansys.com/.

